# 5-Methyl-1,3-diphenyl-*N*-(5-phenyl-1,3,4-thia­diazol-2-yl)-1*H*-pyrazole-4-carboxamide

**DOI:** 10.1107/S1600536813028766

**Published:** 2013-11-06

**Authors:** Chandra, S. S. Mahesh, N. Srikantamurthy, K. B. Umesha, K. Palani, M. Mahendra

**Affiliations:** a Department of Studies in Physics, Manasagangotri, University of Mysore, Mysore 570 006, India; b Department of Physics, Acharya Institute of Technology, Bangalore 560 090, India; c Department of Chemistry, Yuvaraja’s College, University of Mysore, Mysore 570 005, India; d SER-CAT, APS, Argonne National Laboratory, Argonne, IL-60439, USA

## Abstract

The asymmetric unit of the title compound C_25_H_19_N_5_OS, contains two mol­ecules, *A* and *B*. In mol­ecule *A*, the dihedral angles between the pyrazole ring and the C-bound phenyl group, the N-bound phenyl group and the thia­diazole ring are 32.30 (14), 52.25 (14) and 34.94 (12)°, respectively. The corresponding angles in mol­ecule *B* are 33.32 (14), 50.67 (15), and 70.30 (12)°, respectively. In the crystal, the *A* and *B* mol­ecules are linked by pairs of N—H⋯N hydrogen bonds, generating *R*_2_^2^(8) loops. This dimer linkage is reinforced by two C—H⋯O hydrogen bonds and one C—H⋯N hydrogen bond.

## Related literature

For the synthesis, see: Shridevi Doddaramappa *et al.* (2013 ▶). For a related structure, see: Chandra *et al.* (2012 ▶).
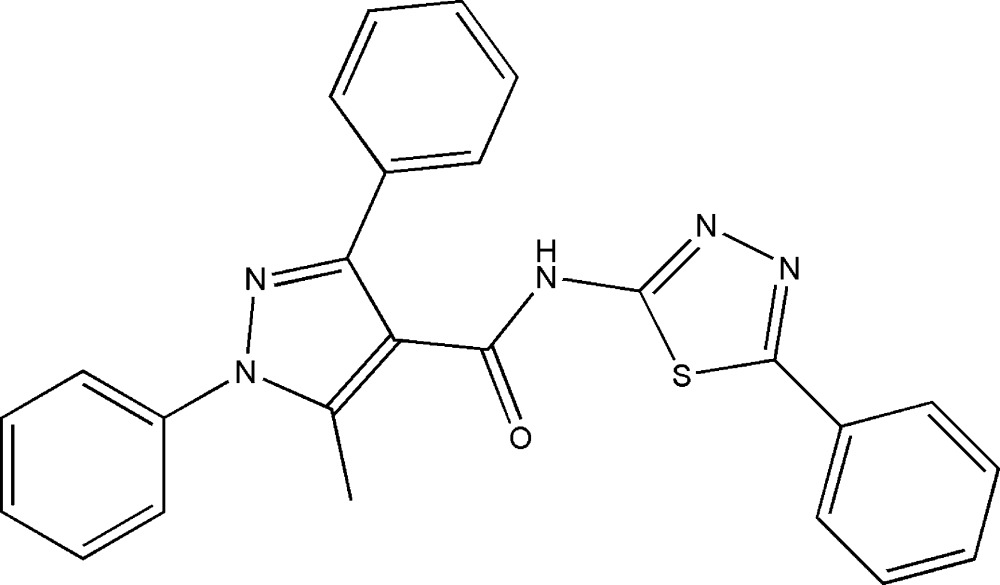


## Experimental

### 

#### Crystal data

C_25_H_19_N_5_OS
*M* *_r_* = 437.52
Triclinic, 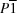

*a* = 10.7039 (14) Å
*b* = 12.9341 (17) Å
*c* = 17.478 (2) Å
α = 77.343 (2)°
β = 82.865 (2)°
γ = 70.373 (2)°
*V* = 2220.4 (5) Å^3^
*Z* = 4
Mo *K*α radiation
μ = 0.17 mm^−1^
*T* = 273 K
0.30 × 0.25 × 0.20 mm

#### Data collection

Bruker APEXII CCD area-detector diffractometer
18748 measured reflections
7768 independent reflections
5447 reflections with *I* > 2σ(*I*)
*R*_int_ = 0.029

#### Refinement

*R*[*F* ^2^ > 2σ(*F* ^2^)] = 0.049
*wR*(*F* ^2^) = 0.133
*S* = 1.02
7768 reflections
579 parameters
H-atom parameters constrained
Δρ_max_ = 0.20 e Å^−3^
Δρ_min_ = −0.19 e Å^−3^

### 

Data collection: *APEX2* (Bruker, 2009 ▶); cell refinement: *SAINT* (Bruker, 2009 ▶); data reduction: *SAINT*; program(s) used to solve structure: *SHELXS97* (Sheldrick, 2008 ▶); program(s) used to refine structure: *SHELXL97* (Sheldrick, 2008 ▶); molecular graphics: *PLATON* (Spek, 2009 ▶); software used to prepare material for publication: *SHELXL97*.

## Supplementary Material

Crystal structure: contains datablock(s) global, I. DOI: 10.1107/S1600536813028766/hb7148sup1.cif

Structure factors: contains datablock(s) I. DOI: 10.1107/S1600536813028766/hb7148Isup2.hkl

Supplementary material file. DOI: 10.1107/S1600536813028766/hb7148Isup3.cml

Additional supplementary materials:  crystallographic information; 3D view; checkCIF report

## Figures and Tables

**Table 1:** Hydrogen-bond geometry (Å, °)

*D*—H⋯*A*	*D*—H	H⋯*A*	*D*⋯*A*	*D*—H⋯*A*
N3—H21⋯N9*B*	0.86	2.13	2.916 (3)	151
N8*B*—H53⋯N4*A*	0.86	2.07	2.902 (3)	163
C4*A*—H6*B*⋯O1*A*	0.96	2.42	3.107 (3)	128
C29*B*—H38*C*⋯N5*A*	0.96	2.59	3.443 (3)	148
C37*B*—H46⋯O2*B*	0.93	2.41	3.184 (3)	141

**Fig. 1.**
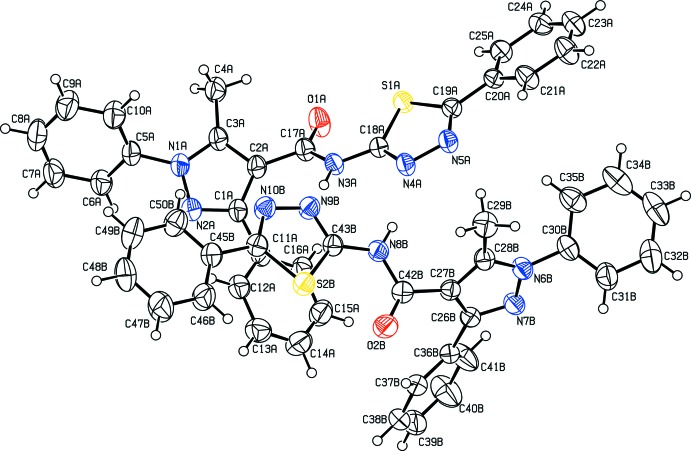
Perspective diagram of (I) with 50% probability displacement ellipsoids.

**Fig. 2.**
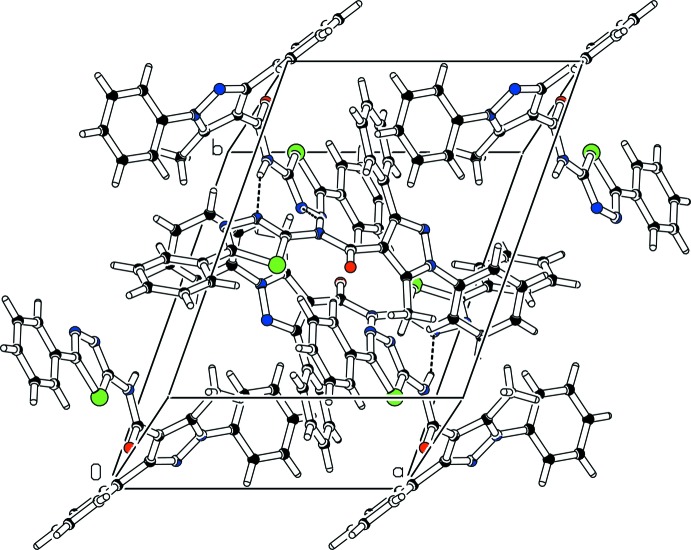
Packing diagram of the molecule viewed down the 'c' axis.

## supplementary crystallographic information

### 1. Comment

As part of our ongoing studies of pyrazole derivatives (Shridevi Doddaramappa, *et al.*, 2013), the title compound, (I), was prepared and characterized by single-crystal X-ray diffraction. It shows potential anticancer activity against the aurora kinase enzyme: the full results will be reported elsewhere.

In the molecular structure of the title compound (Fig. 1), two molecules (A and B) present in an asymmetric unit have heterocyclic ring moieties exhibiting planar conformation, with their maximum deviations found on ring planes from N1A and C19A are 0.011 (2) Å and 0.005 (2) Å, respectively. The bond lengths are comparable to a related structure (Chandra *et al.*, 2012).

In the molecules A and B, pyrazole moiety makes a dihedral angle of 34.94 (12)°, 76.83 (12)°, 50.67 (13)° and 33.32 (14)° with the phenyl rings (C5A–C10A, C11A–C16A and C30B–C35B, C36B–C41B), respectively. Thiadiazole moiety makes a dihedral angle of 58.01 (13)°, 68.82 (13)°, 79.35 (13)° and 70.30 (12)° with pyrazole and benzene rings, for A and B, respectively.

The crystal structure features C—H···N and C—H···O hydrogen bonds. The packing diagram of the molecules exhibit dimers with stacking feature when viewed down the *c* axis is shown in Fig. 2.

### 2. Experimental

A solution of pyrazole-4-carboxylic acid (1 mmol) in dry dichloromethane (5 ml) was cooled to 0° C and added ethyl-(*N*',*N*'-dimethylamino)propylcarbodiimide hydrochloride (EDC·HCl, 1.2 mmol) and HOBt (1.2 mmol). To this reaction mixture 2-amino-5-phenyl-1,3,4- thiadiazole (1 mmol) was added and stirred at room temperature for 6–8 h. After completion of the reaction, the reaction mixture was extracted with ethyl acetate (2 × 25 ml) and the combined organic phase was washed with brine, dried over anhydrous sodium sulfate. Ethyl acetate was distilled off and the residue thus obtained was purified by recrystallization in EtOH to get the title compound as colourless blocks.

### 3. Refinement

H atoms were placed at idealized positions and allowed to ride on their parent atoms with N–H distance is equal to 0.86 Å and C–H distances in the range of 0.93 to 0.96 Å; *U*_iso_(H) = 1.2–1.5*U*_eq_(carrier atom) for all H atoms.

### Crystal data

**Table:**

C_25_H_19_N_5_OS	*Z* = 4
*M**_r_* = 437.52	*F*(000) = 912
Triclinic, *P*1	*D*_x_ = 1.309 Mg m^−^^3^
Hall symbol: -P 1	Mo *K*α radiation, λ = 0.71073 Å
*a* = 10.7039 (14) Å	Cell parameters from 7768 reflections
*b* = 12.9341 (17) Å	θ = 1.7–25.0°
*c* = 17.478 (2) Å	µ = 0.17 mm^−^^1^
α = 77.343 (2)°	*T* = 273 K
β = 82.865 (2)°	Block, colourless
γ = 70.373 (2)°	0.30 × 0.25 × 0.20 mm
*V* = 2220.4 (5) Å^3^	

### Data collection

**Table:**

Bruker APEXII CCD area-detector diffractometer	*R*_int_ = 0.029
ω and φ scans	θ_max_ = 25.0°, θ_min_ = 1.7°
18748 measured reflections	*h* = −12→12
7768 independent reflections	*k* = −15→15
5447 reflections with *I* > 2σ(*I*)	*l* = −20→20

### Refinement

**Table:**

Refinement on *F*^2^	Primary atom site location: structure-invariant direct methods
Least-squares matrix: full	Secondary atom site location: difference Fourier map
*R*[*F*^2^ > 2σ(*F*^2^)] = 0.049	Hydrogen site location: inferred from neighbouring sites
*wR*(*F*^2^) = 0.133	H-atom parameters constrained
*S* = 1.02	*w* = 1/[σ^2^(*F*_o_^2^) + (0.0689*P*)^2^] where *P* = (*F*_o_^2^ + 2*F*_c_^2^)/3
7768 reflections	(Δ/σ)_max_ < 0.001
579 parameters	Δρ_max_ = 0.20 e Å^−^^3^
0 restraints	Δρ_min_ = −0.19 e Å^−^^3^

### Special details

**Table:**

Geometry. Bond distances, angles *etc*. have been calculated using the rounded fractional coordinates. All su's are estimated from the variances of the (full) variance-covariance matrix. The cell e.s.d.'s are taken into account in the estimation of distances, angles and torsion angles
Refinement. Refinement on *F*^2^ for ALL reflections except those flagged by the user for potential systematic errors. Weighted *R*-factors *wR* and all goodnesses of fit *S* are based on *F*^2^, conventional *R*-factors *R* are based on *F*, with *F* set to zero for negative *F*^2^. The observed criterion of *F*^2^ > σ(*F*^2^) is used only for calculating -*R*-factor-obs *etc*. and is not relevant to the choice of reflections for refinement. *R*-factors based on *F*^2^ are statistically about twice as large as those based on *F*, and *R*-factors based on ALL data will be even larger.

### Fractional atomic coordinates and isotropic or equivalent isotropic displacement parameters (Å^2^)

**Table:**

	*x*	*y*	*z*	*U*_iso_*/*U*_eq_	
S1A	0.77966 (6)	0.57443 (5)	0.11413 (3)	0.0486 (2)	
O1A	0.52592 (17)	0.57319 (15)	0.14214 (10)	0.0696 (7)	
N1A	0.24119 (19)	0.51919 (16)	0.32617 (11)	0.0509 (7)	
N2A	0.3029 (2)	0.40699 (16)	0.34827 (11)	0.0528 (7)	
N3	0.66483 (18)	0.44976 (15)	0.23031 (10)	0.0459 (7)	
N4A	0.89440 (19)	0.40837 (15)	0.21844 (10)	0.0479 (7)	
N5A	0.99500 (19)	0.44439 (16)	0.17621 (11)	0.0491 (7)	
C1A	0.4177 (2)	0.38576 (19)	0.30667 (13)	0.0470 (8)	
C2A	0.4297 (2)	0.48482 (19)	0.25603 (13)	0.0471 (8)	
C3A	0.3132 (2)	0.5683 (2)	0.26966 (13)	0.0503 (8)	
C4A	0.2660 (3)	0.6889 (2)	0.23304 (15)	0.0661 (10)	
C5A	0.1144 (2)	0.5672 (2)	0.36390 (13)	0.0520 (9)	
C6A	0.0175 (3)	0.5179 (2)	0.36746 (15)	0.0643 (10)	
C7A	−0.1019 (3)	0.5587 (3)	0.40836 (18)	0.0775 (11)	
C8A	−0.1239 (3)	0.6480 (3)	0.44329 (18)	0.0830 (14)	
C9A	−0.0278 (3)	0.6974 (3)	0.43862 (17)	0.0834 (12)	
C10A	0.0925 (3)	0.6563 (2)	0.39990 (16)	0.0691 (11)	
C11A	0.5030 (2)	0.26915 (19)	0.31428 (14)	0.0474 (8)	
C12A	0.5017 (3)	0.1949 (2)	0.38506 (15)	0.0585 (10)	
C13A	0.5736 (3)	0.0835 (2)	0.39154 (18)	0.0745 (11)	
C14A	0.6473 (3)	0.0437 (3)	0.3285 (2)	0.0820 (13)	
C15A	0.6500 (3)	0.1148 (2)	0.25814 (19)	0.0734 (12)	
C16A	0.5786 (2)	0.2267 (2)	0.25111 (15)	0.0580 (9)	
C17A	0.5401 (2)	0.5061 (2)	0.20410 (14)	0.0495 (8)	
C18A	0.7777 (2)	0.46898 (18)	0.19296 (12)	0.0417 (7)	
C19A	0.9504 (2)	0.53004 (18)	0.12061 (13)	0.0438 (8)	
C20A	1.0379 (2)	0.58313 (18)	0.06721 (12)	0.0440 (8)	
C21A	1.1676 (3)	0.5616 (2)	0.08475 (15)	0.0591 (9)	
C22A	1.2503 (3)	0.6097 (2)	0.03406 (16)	0.0716 (11)	
C23A	1.2041 (3)	0.6815 (2)	−0.03456 (16)	0.0662 (11)	
C24A	1.0754 (3)	0.7045 (2)	−0.05280 (14)	0.0587 (10)	
C25A	0.9926 (3)	0.65537 (19)	−0.00233 (13)	0.0532 (8)	
S2B	0.81627 (7)	0.14175 (5)	0.48058 (3)	0.0537 (2)	
O2B	0.98933 (19)	0.01874 (14)	0.37824 (9)	0.0678 (7)	
N6B	1.2354 (2)	0.11455 (16)	0.16431 (11)	0.0509 (7)	
N7B	1.1838 (2)	0.04476 (16)	0.14004 (11)	0.0523 (7)	
N8B	0.91824 (19)	0.20392 (15)	0.33307 (10)	0.0487 (7)	
N9B	0.7403 (2)	0.33063 (16)	0.38824 (10)	0.0525 (7)	
N10B	0.6575 (2)	0.34209 (16)	0.45472 (11)	0.0539 (7)	
C26B	1.0909 (2)	0.02892 (18)	0.19482 (13)	0.0458 (8)	
C27B	1.0831 (2)	0.08937 (18)	0.25492 (12)	0.0447 (8)	
C28B	1.1771 (2)	0.14337 (19)	0.23367 (13)	0.0482 (8)	
C29B	1.2217 (3)	0.2113 (2)	0.27610 (15)	0.0650 (10)	
C30B	1.3427 (3)	0.1439 (2)	0.11818 (14)	0.0557 (9)	
C31B	1.4489 (3)	0.0614 (3)	0.09391 (16)	0.0724 (11)	
C32B	1.5488 (3)	0.0908 (3)	0.04525 (18)	0.0907 (14)	
C33B	1.5409 (4)	0.2004 (4)	0.02129 (18)	0.0925 (16)	
C34B	1.4365 (4)	0.2816 (3)	0.04664 (18)	0.0860 (16)	
C35B	1.3356 (3)	0.2547 (2)	0.09550 (16)	0.0695 (11)	
C36B	1.0101 (2)	−0.03876 (18)	0.18330 (14)	0.0481 (8)	
C37B	0.9612 (3)	−0.10363 (19)	0.24491 (15)	0.0565 (9)	
C38B	0.8893 (3)	−0.1677 (2)	0.23079 (18)	0.0686 (11)	
C39B	0.8640 (3)	−0.1672 (2)	0.1564 (2)	0.0827 (12)	
C40B	0.9107 (4)	−0.1026 (3)	0.09536 (19)	0.1041 (18)	
C41B	0.9826 (3)	−0.0384 (3)	0.10839 (16)	0.0852 (13)	
C42B	0.9947 (2)	0.09754 (19)	0.32675 (13)	0.0474 (8)	
C43B	0.8266 (2)	0.23077 (19)	0.39388 (12)	0.0450 (8)	
C44B	0.6846 (2)	0.25151 (19)	0.50738 (13)	0.0484 (8)	
C45B	0.6065 (3)	0.2402 (2)	0.58222 (13)	0.0524 (9)	
C46B	0.6323 (3)	0.1389 (2)	0.63370 (15)	0.0765 (11)	
C47B	0.5571 (4)	0.1274 (3)	0.70274 (17)	0.0940 (14)	
C48B	0.4564 (3)	0.2173 (3)	0.72158 (17)	0.0841 (13)	
C49B	0.4298 (3)	0.3172 (3)	0.67167 (17)	0.0773 (11)	
C50B	0.5042 (3)	0.3306 (2)	0.60176 (15)	0.0676 (11)	
H6A	0.17840	0.70790	0.21520	0.0990*	
H6B	0.32520	0.70370	0.18920	0.0990*	
H6C	0.26380	0.73310	0.27100	0.0990*	
H8	0.03230	0.45780	0.34260	0.0770*	
H9	−0.16740	0.52520	0.41200	0.0930*	
H10	−0.20460	0.67550	0.47050	0.1000*	
H11	−0.04420	0.75920	0.46180	0.1000*	
H12	0.15870	0.68860	0.39810	0.0830*	
H14	0.45160	0.22100	0.42840	0.0700*	
H15	0.57220	0.03490	0.43930	0.0890*	
H16	0.69560	−0.03180	0.33340	0.0980*	
H17	0.70010	0.08760	0.21510	0.0880*	
H18	0.58100	0.27460	0.20320	0.0700*	
H21	0.67280	0.39960	0.27240	0.0550*	
H28	1.19930	0.51400	0.13130	0.0710*	
H29	1.33780	0.59380	0.04620	0.0860*	
H30	1.26010	0.71440	−0.06850	0.0790*	
H31	1.04400	0.75300	−0.09910	0.0700*	
H32	0.90560	0.67080	−0.01500	0.0640*	
H38A	1.31680	0.19090	0.27100	0.0970*	
H38B	1.19340	0.19780	0.33060	0.0970*	
H38C	1.18360	0.28920	0.25410	0.0970*	
H40	1.45380	−0.01330	0.10990	0.0870*	
H41	1.62190	0.03540	0.02880	0.1090*	
H42	1.60730	0.21950	−0.01250	0.1110*	
H43	1.43280	0.35600	0.03100	0.1030*	
H44	1.26400	0.31040	0.11280	0.0840*	
H46	0.97670	−0.10430	0.29620	0.0680*	
H47	0.85770	−0.21180	0.27280	0.0820*	
H48	0.81530	−0.21050	0.14750	0.0990*	
H49	0.89380	−0.10180	0.04430	0.1250*	
H50	1.01310	0.00590	0.06590	0.1020*	
H53	0.92810	0.25770	0.29660	0.0580*	
H60	0.70160	0.07760	0.62150	0.0920*	
H61	0.57490	0.05850	0.73660	0.1130*	
H62	0.40610	0.21000	0.76860	0.1010*	
H63	0.36030	0.37790	0.68460	0.0930*	
H64	0.48530	0.39990	0.56820	0.0810*	

### Atomic displacement parameters (Å^2^)

**Table:**

	*U*^11^	*U*^22^	*U*^33^	*U*^12^	*U*^13^	*U*^23^
S1A	0.0405 (4)	0.0475 (3)	0.0524 (4)	−0.0139 (3)	−0.0025 (3)	0.0014 (3)
O1A	0.0463 (11)	0.0901 (13)	0.0533 (10)	−0.0160 (10)	−0.0047 (8)	0.0176 (10)
N1A	0.0368 (12)	0.0560 (12)	0.0572 (12)	−0.0135 (10)	0.0044 (10)	−0.0114 (10)
N2A	0.0436 (13)	0.0564 (13)	0.0590 (12)	−0.0181 (10)	0.0026 (10)	−0.0117 (10)
N3	0.0370 (12)	0.0490 (11)	0.0457 (11)	−0.0141 (9)	−0.0006 (9)	0.0031 (9)
N4A	0.0367 (12)	0.0549 (12)	0.0480 (11)	−0.0161 (10)	0.0002 (9)	−0.0001 (9)
N5A	0.0383 (12)	0.0568 (12)	0.0491 (11)	−0.0169 (10)	−0.0009 (9)	−0.0019 (9)
C1A	0.0371 (14)	0.0562 (15)	0.0488 (13)	−0.0160 (12)	0.0014 (11)	−0.0127 (11)
C2A	0.0359 (14)	0.0560 (14)	0.0469 (13)	−0.0129 (12)	−0.0020 (11)	−0.0073 (11)
C3A	0.0382 (15)	0.0602 (15)	0.0510 (14)	−0.0165 (13)	0.0003 (11)	−0.0078 (12)
C4A	0.0471 (17)	0.0640 (17)	0.0733 (17)	−0.0092 (13)	0.0051 (13)	−0.0028 (14)
C5A	0.0379 (15)	0.0636 (16)	0.0512 (14)	−0.0147 (13)	0.0033 (11)	−0.0098 (12)
C6A	0.0460 (17)	0.0695 (17)	0.0750 (18)	−0.0179 (14)	0.0012 (14)	−0.0122 (14)
C7A	0.0440 (18)	0.088 (2)	0.091 (2)	−0.0201 (16)	0.0068 (16)	−0.0051 (18)
C8A	0.052 (2)	0.102 (3)	0.078 (2)	−0.0100 (19)	0.0187 (16)	−0.0174 (19)
C9A	0.073 (2)	0.096 (2)	0.080 (2)	−0.0194 (19)	0.0174 (18)	−0.0381 (18)
C10A	0.0551 (19)	0.086 (2)	0.0730 (18)	−0.0249 (16)	0.0054 (15)	−0.0305 (16)
C11A	0.0368 (14)	0.0545 (14)	0.0565 (14)	−0.0194 (12)	−0.0008 (11)	−0.0151 (12)
C12A	0.0505 (17)	0.0624 (17)	0.0636 (16)	−0.0209 (14)	0.0003 (13)	−0.0111 (13)
C13A	0.070 (2)	0.0608 (18)	0.085 (2)	−0.0195 (16)	−0.0054 (17)	0.0006 (16)
C14A	0.068 (2)	0.0597 (18)	0.113 (3)	−0.0102 (16)	0.000 (2)	−0.0242 (19)
C15A	0.063 (2)	0.073 (2)	0.087 (2)	−0.0178 (16)	0.0095 (16)	−0.0350 (17)
C16A	0.0479 (16)	0.0666 (17)	0.0635 (16)	−0.0202 (14)	0.0009 (13)	−0.0197 (13)
C17A	0.0414 (15)	0.0566 (14)	0.0475 (14)	−0.0156 (12)	−0.0015 (11)	−0.0040 (12)
C18A	0.0385 (14)	0.0443 (12)	0.0424 (12)	−0.0143 (11)	0.0008 (10)	−0.0083 (10)
C19A	0.0433 (14)	0.0446 (13)	0.0457 (13)	−0.0168 (11)	−0.0011 (11)	−0.0094 (11)
C20A	0.0438 (15)	0.0434 (12)	0.0496 (13)	−0.0196 (11)	0.0011 (11)	−0.0117 (10)
C21A	0.0543 (17)	0.0670 (16)	0.0601 (16)	−0.0306 (14)	−0.0089 (13)	0.0003 (13)
C22A	0.0560 (19)	0.091 (2)	0.0782 (19)	−0.0424 (17)	−0.0053 (15)	−0.0063 (16)
C23A	0.069 (2)	0.0742 (18)	0.0649 (17)	−0.0431 (16)	0.0078 (15)	−0.0071 (14)
C24A	0.069 (2)	0.0554 (15)	0.0523 (15)	−0.0265 (14)	−0.0017 (13)	−0.0018 (12)
C25A	0.0500 (16)	0.0549 (14)	0.0536 (14)	−0.0189 (13)	−0.0047 (12)	−0.0034 (12)
S2B	0.0580 (4)	0.0537 (4)	0.0400 (3)	−0.0105 (3)	−0.0003 (3)	−0.0026 (3)
O2B	0.0840 (14)	0.0523 (10)	0.0497 (10)	−0.0100 (10)	0.0055 (9)	0.0026 (8)
N6B	0.0495 (13)	0.0572 (12)	0.0518 (12)	−0.0250 (11)	0.0016 (10)	−0.0118 (10)
N7B	0.0555 (14)	0.0553 (12)	0.0515 (12)	−0.0261 (11)	0.0042 (10)	−0.0120 (10)
N8B	0.0518 (13)	0.0473 (11)	0.0406 (10)	−0.0137 (10)	0.0008 (9)	−0.0002 (9)
N9B	0.0555 (14)	0.0518 (12)	0.0420 (11)	−0.0106 (11)	0.0018 (10)	−0.0047 (9)
N10B	0.0561 (14)	0.0562 (12)	0.0444 (11)	−0.0126 (11)	0.0021 (10)	−0.0105 (10)
C26B	0.0481 (15)	0.0430 (13)	0.0449 (13)	−0.0149 (11)	−0.0002 (11)	−0.0063 (10)
C27B	0.0438 (15)	0.0433 (12)	0.0425 (12)	−0.0098 (11)	−0.0035 (11)	−0.0046 (10)
C28B	0.0430 (15)	0.0532 (14)	0.0471 (13)	−0.0141 (12)	−0.0046 (11)	−0.0078 (11)
C29B	0.0605 (18)	0.0777 (18)	0.0663 (17)	−0.0273 (15)	−0.0106 (14)	−0.0209 (14)
C30B	0.0530 (17)	0.0725 (18)	0.0487 (14)	−0.0323 (15)	−0.0037 (12)	−0.0058 (13)
C31B	0.070 (2)	0.085 (2)	0.0674 (18)	−0.0339 (18)	0.0174 (15)	−0.0222 (16)
C32B	0.073 (2)	0.131 (3)	0.083 (2)	−0.050 (2)	0.0238 (18)	−0.039 (2)
C33B	0.094 (3)	0.140 (3)	0.072 (2)	−0.082 (3)	0.0101 (19)	−0.016 (2)
C34B	0.094 (3)	0.103 (3)	0.077 (2)	−0.065 (2)	−0.012 (2)	0.0075 (19)
C35B	0.065 (2)	0.0750 (19)	0.0731 (18)	−0.0354 (16)	−0.0157 (15)	0.0036 (15)
C36B	0.0483 (15)	0.0417 (13)	0.0556 (14)	−0.0168 (11)	0.0073 (12)	−0.0134 (11)
C37B	0.0570 (17)	0.0470 (14)	0.0613 (15)	−0.0164 (13)	0.0075 (13)	−0.0082 (12)
C38B	0.0621 (19)	0.0520 (16)	0.091 (2)	−0.0262 (14)	0.0218 (16)	−0.0141 (15)
C39B	0.092 (2)	0.081 (2)	0.102 (2)	−0.0551 (19)	0.029 (2)	−0.0482 (19)
C40B	0.148 (4)	0.136 (3)	0.076 (2)	−0.100 (3)	0.022 (2)	−0.047 (2)
C41B	0.127 (3)	0.106 (2)	0.0583 (17)	−0.085 (2)	0.0142 (17)	−0.0237 (16)
C42B	0.0473 (15)	0.0507 (14)	0.0413 (13)	−0.0129 (12)	−0.0072 (11)	−0.0043 (11)
C43B	0.0450 (15)	0.0500 (14)	0.0387 (12)	−0.0142 (12)	−0.0022 (11)	−0.0073 (10)
C44B	0.0515 (16)	0.0536 (14)	0.0397 (12)	−0.0157 (12)	−0.0042 (11)	−0.0090 (11)
C45B	0.0513 (16)	0.0632 (16)	0.0434 (13)	−0.0184 (13)	−0.0024 (12)	−0.0116 (12)
C46B	0.081 (2)	0.0732 (19)	0.0565 (17)	−0.0116 (17)	0.0165 (15)	−0.0057 (14)
C47B	0.100 (3)	0.097 (2)	0.0641 (19)	−0.025 (2)	0.0201 (19)	0.0038 (17)
C48B	0.071 (2)	0.123 (3)	0.0550 (17)	−0.034 (2)	0.0165 (16)	−0.0164 (19)
C49B	0.057 (2)	0.103 (2)	0.0642 (18)	−0.0075 (18)	0.0030 (15)	−0.0323 (18)
C50B	0.065 (2)	0.0756 (19)	0.0543 (16)	−0.0101 (16)	−0.0016 (14)	−0.0168 (14)

### Geometric parameters (Å, º)

**Table:**

S1A—C18A	1.717 (2)	C8A—H10	0.9300
S1A—C19A	1.732 (2)	C9A—H11	0.9300
S2B—C43B	1.707 (2)	C10A—H12	0.9300
S2B—C44B	1.733 (2)	C12A—H14	0.9300
O1A—C17A	1.220 (3)	C13A—H15	0.9300
O2B—C42B	1.215 (3)	C14A—H16	0.9300
N1A—C5A	1.433 (3)	C15A—H17	0.9300
N1A—N2A	1.366 (3)	C16A—H18	0.9300
N1A—C3A	1.356 (3)	C21A—H28	0.9300
N2A—C1A	1.324 (3)	C22A—H29	0.9300
N3—C17A	1.370 (3)	C23A—H30	0.9300
N3—C18A	1.372 (3)	C24A—H31	0.9300
N4A—C18A	1.304 (3)	C25A—H32	0.9300
N4A—N5A	1.382 (3)	C26B—C27B	1.420 (3)
N5A—C19A	1.299 (3)	C26B—C36B	1.478 (3)
N3—H21	0.8600	C27B—C42B	1.476 (3)
N6B—C28B	1.361 (3)	C27B—C28B	1.380 (3)
N6B—N7B	1.364 (3)	C28B—C29B	1.485 (4)
N6B—C30B	1.429 (4)	C30B—C31B	1.369 (4)
N7B—C26B	1.327 (3)	C30B—C35B	1.379 (4)
N8B—C42B	1.365 (3)	C31B—C32B	1.385 (5)
N8B—C43B	1.373 (3)	C32B—C33B	1.364 (6)
N9B—C43B	1.303 (3)	C33B—C34B	1.357 (6)
N9B—N10B	1.374 (3)	C34B—C35B	1.382 (5)
N10B—C44B	1.292 (3)	C36B—C41B	1.376 (4)
N8B—H53	0.8600	C36B—C37B	1.377 (4)
C1A—C2A	1.424 (3)	C37B—C38B	1.380 (4)
C1A—C11A	1.464 (3)	C38B—C39B	1.359 (5)
C2A—C17A	1.462 (3)	C39B—C40B	1.358 (5)
C2A—C3A	1.383 (3)	C40B—C41B	1.375 (5)
C3A—C4A	1.487 (3)	C44B—C45B	1.469 (3)
C5A—C6A	1.377 (4)	C45B—C50B	1.380 (4)
C5A—C10A	1.371 (4)	C45B—C46B	1.380 (3)
C6A—C7A	1.381 (5)	C46B—C47B	1.374 (4)
C7A—C8A	1.361 (5)	C47B—C48B	1.364 (5)
C8A—C9A	1.368 (5)	C48B—C49B	1.354 (5)
C9A—C10A	1.371 (5)	C49B—C50B	1.384 (4)
C11A—C12A	1.392 (3)	C29B—H38A	0.9600
C11A—C16A	1.386 (3)	C29B—H38B	0.9600
C12A—C13A	1.374 (4)	C29B—H38C	0.9600
C13A—C14A	1.364 (5)	C31B—H40	0.9300
C14A—C15A	1.368 (5)	C32B—H41	0.9300
C15A—C16A	1.377 (4)	C33B—H42	0.9300
C19A—C20A	1.468 (3)	C34B—H43	0.9300
C20A—C25A	1.389 (3)	C35B—H44	0.9300
C20A—C21A	1.381 (4)	C37B—H46	0.9300
C21A—C22A	1.375 (4)	C38B—H47	0.9300
C22A—C23A	1.377 (4)	C39B—H48	0.9300
C23A—C24A	1.371 (5)	C40B—H49	0.9300
C24A—C25A	1.381 (4)	C41B—H50	0.9300
C4A—H6C	0.9600	C46B—H60	0.9300
C4A—H6B	0.9600	C47B—H61	0.9300
C4A—H6A	0.9600	C48B—H62	0.9300
C6A—H8	0.9300	C49B—H63	0.9300
C7A—H9	0.9300	C50B—H64	0.9300
			
S1A···O1A	2.704 (2)	C23A···H42^vii^	3.0000
S1A···N5A	2.557 (2)	C26B···H31^i^	2.9400
S1A···C34B^i^	3.626 (4)	C27B···H46	3.0000
S2B···O2B^ii^	3.2950 (19)	C28B···H44	2.9800
S2B···N10B	2.555 (2)	C28B···H61^ii^	3.0400
S2B···O2B	2.7580 (19)	C28B···H53	2.8000
S2B···C13A	3.545 (3)	C29B···H44	2.9100
S1A···H48^iii^	3.1100	C29B···H53	2.9900
S1A···H32	2.7600	C30B···H38A	2.8300
S2B···H60	2.7000	C31B···H61^ii^	3.0500
O1A···S1A	2.704 (2)	C31B···H41^ix^	2.9900
O1A···C4A	3.107 (3)	C32B···H40^ix^	3.1000
O2B···S2B	2.7580 (19)	C33B···H30^vii^	2.9700
O2B···S2B^ii^	3.2950 (19)	C35B···H32^i^	2.8700
O2B···C37B	3.184 (3)	C35B···H38C	3.0800
O1A···H6B	2.4200	C35B···H38A	3.0000
O1A···H29^iv^	2.6900	C37B···H16	3.0200
O2B···H46	2.4100	C37B···H17	3.0600
N2A···C29B^iv^	3.450 (3)	C37B···H6A^x^	2.8400
N3···C11A	3.346 (3)	C38B···H16	2.9400
N3···C16A	3.249 (3)	C38B···H6A^x^	2.9800
N3···N9B	2.916 (3)	C40B···H50^xi^	2.9600
N4A···S1A	2.5522 (19)	C41B···H50^xi^	2.9800
N4A···N8B	2.902 (3)	C42B···H46	2.8500
N5A···S1A	2.557 (2)	C42B···H38B	2.8500
N5A···C29B	3.443 (3)	C43B···H21	2.9000
N8B···N4A	2.902 (3)	C44B···H12^v^	2.8700
N8B···C29B	3.304 (4)	C45B···H12^v^	3.0300
N9B···N3	2.916 (3)	H6A···C38B^vi^	2.9800
N9B···S2B	2.549 (2)	H6A···C5A	2.9800
N9B···C11A	3.373 (3)	H6A···C37B^vi^	2.8400
N10B···S2B	2.555 (2)	H6B···O1A	2.4200
N10B···C12A	3.397 (4)	H6B···C17A	2.7900
N2A···H14	2.6100	H6C···C10A	2.9400
N2A···H8	2.7600	H6C···H12	2.4100
N3···H63^v^	2.8600	H8···N2A	2.7600
N3···H18	2.8500	H12···C44B^v^	2.8700
N4A···H53	2.0700	H12···C45B^v^	3.0300
N5A···H28	2.6100	H12···H6C	2.4100
N5A···H38C	2.5900	H12···C3A	3.0100
N6B···H61^ii^	2.8700	H12···C4A	2.9700
N7B···H31^i^	2.9400	H14···N2A	2.6100
N7B···H40	2.7400	H16···C38B	2.9400
N7B···H50	2.6000	H16···C37B	3.0200
N8B···H38B	2.9100	H17···C37B	3.0600
N9B···H21	2.1300	H18···C17A	2.8800
N10B···H64	2.5900	H18···N3	2.8500
C4A···C10A	3.282 (4)	H18···C2A	2.9400
C4A···C37B^vi^	3.476 (4)	H21···C1A	2.7800
C4A···O1A	3.107 (3)	H21···C11A	2.8100
C10A···C4A	3.282 (4)	H21···N9B	2.1300
C11A···N9B	3.373 (3)	H21···C43B	2.9000
C11A···N3	3.346 (3)	H21···C16A	2.8500
C11A···C29B^iv^	3.508 (4)	H28···N5A	2.6100
C12A···N10B	3.397 (4)	H28···H44	2.5700
C12A···C44B	3.410 (4)	H29···O1A^viii^	2.6900
C13A···S2B	3.545 (3)	H30···C33B^vii^	2.9700
C14A···C37B	3.571 (5)	H30···H42^vii^	2.5700
C15A···C37B	3.592 (4)	H31···N7B^i^	2.9400
C16A···C17A	3.419 (4)	H31···C26B^i^	2.9400
C16A···N3	3.249 (3)	H32···H44^i^	2.5600
C17A···C16A	3.419 (4)	H32···S1A	2.7600
C17A···C49B^v^	3.590 (4)	H32···C35B^i^	2.8700
C18A···C24A^i^	3.548 (3)	H38A···C11A^viii^	2.7600
C19A···C24A^i^	3.592 (3)	H38A···C12A^viii^	3.0000
C19A···C25A^i^	3.358 (3)	H38A···C16A^viii^	2.9600
C23A···C33B^vii^	3.599 (6)	H38A···C30B	2.8300
C24A···C18A^i^	3.548 (3)	H38A···C35B	3.0000
C24A···C19A^i^	3.592 (3)	H38B···N8B	2.9100
C25A···C19A^i^	3.358 (3)	H38B···C42B	2.8500
C29B···C35B	3.239 (4)	H38C···N5A	2.5900
C29B···N2A^viii^	3.450 (3)	H38C···C35B	3.0800
C29B···N5A	3.443 (3)	H38C···H44	2.5000
C29B···C11A^viii^	3.508 (4)	H40···N7B	2.7400
C29B···N8B	3.304 (4)	H40···C32B^ix^	3.1000
C31B···C32B^ix^	3.441 (5)	H41···C31B^ix^	2.9900
C32B···C31B^ix^	3.441 (5)	H42···C22A^vii^	3.0200
C32B···C32B^ix^	3.577 (5)	H42···C23A^vii^	3.0000
C33B···C23A^vii^	3.599 (6)	H42···H30^vii^	2.5700
C34B···S1A^i^	3.626 (4)	H44···C21A	3.0100
C35B···C29B	3.239 (4)	H44···C28B	2.9800
C37B···C15A	3.592 (4)	H44···C29B	2.9100
C37B···C14A	3.571 (5)	H44···H28	2.5700
C37B···C4A^x^	3.476 (4)	H44···H38C	2.5000
C37B···O2B	3.184 (3)	H44···H32^i^	2.5600
C37B···C42B	3.366 (4)	H46···O2B	2.4100
C42B···C37B	3.366 (4)	H46···C27B	3.0000
C44B···C12A	3.410 (4)	H46···C42B	2.8500
C49B···C17A^v^	3.590 (4)	H48···S1A^xii^	3.1100
C1A···H21	2.7800	H49···H50^xi^	2.3600
C2A···H18	2.9400	H50···N7B	2.6000
C3A···H12	3.0100	H50···C40B^xi^	2.9600
C4A···H12	2.9700	H50···C41B^xi^	2.9800
C5A···H6A	2.9800	H50···H49^xi^	2.3600
C10A···H6C	2.9400	H50···H50^xi^	2.4000
C11A···H21	2.8100	H53···N4A	2.0700
C11A···H38A^iv^	2.7600	H53···C18A	2.9900
C12A···H38A^iv^	3.0000	H53···C28B	2.8000
C16A···H38A^iv^	2.9600	H53···C29B	2.9900
C16A···H21	2.8500	H60···S2B	2.7000
C17A···H6B	2.7900	H61···N6B^ii^	2.8700
C17A···H18	2.8800	H61···C28B^ii^	3.0400
C18A···H53	2.9900	H61···C31B^ii^	3.0500
C21A···H44	3.0100	H63···N3^v^	2.8600
C22A···H42^vii^	3.0200	H64···N10B	2.5900
			
C18A—S1A—C19A	86.67 (11)	C20A—C21A—H28	120.00
C43B—S2B—C44B	86.22 (11)	C22A—C21A—H28	120.00
N2A—N1A—C3A	112.4 (2)	C21A—C22A—H29	120.00
N2A—N1A—C5A	117.77 (19)	C23A—C22A—H29	120.00
C3A—N1A—C5A	129.9 (2)	C22A—C23A—H30	120.00
N1A—N2A—C1A	105.28 (19)	C24A—C23A—H30	120.00
C17A—N3—C18A	123.55 (19)	C23A—C24A—H31	120.00
N5A—N4A—C18A	112.18 (18)	C25A—C24A—H31	120.00
N4A—N5A—C19A	112.3 (2)	C20A—C25A—H32	120.00
C17A—N3—H21	118.00	C24A—C25A—H32	120.00
C18A—N3—H21	118.00	N7B—C26B—C27B	110.2 (2)
N7B—N6B—C28B	112.5 (2)	N7B—C26B—C36B	118.8 (2)
C28B—N6B—C30B	128.8 (2)	C27B—C26B—C36B	130.9 (2)
N7B—N6B—C30B	118.65 (19)	C26B—C27B—C28B	106.27 (19)
N6B—N7B—C26B	105.50 (18)	C28B—C27B—C42B	124.3 (2)
C42B—N8B—C43B	124.31 (19)	C26B—C27B—C42B	129.5 (2)
N10B—N9B—C43B	111.92 (18)	N6B—C28B—C29B	123.4 (2)
N9B—N10B—C44B	112.3 (2)	N6B—C28B—C27B	105.5 (2)
C43B—N8B—H53	118.00	C27B—C28B—C29B	130.9 (2)
C42B—N8B—H53	118.00	N6B—C30B—C35B	120.0 (2)
N2A—C1A—C2A	110.8 (2)	N6B—C30B—C31B	119.2 (2)
N2A—C1A—C11A	117.8 (2)	C31B—C30B—C35B	120.8 (3)
C2A—C1A—C11A	131.2 (2)	C30B—C31B—C32B	119.0 (3)
C1A—C2A—C17A	131.4 (2)	C31B—C32B—C33B	120.5 (4)
C3A—C2A—C17A	123.1 (2)	C32B—C33B—C34B	120.2 (4)
C1A—C2A—C3A	105.3 (2)	C33B—C34B—C35B	120.6 (3)
N1A—C3A—C2A	106.2 (2)	C30B—C35B—C34B	119.0 (3)
C2A—C3A—C4A	130.6 (2)	C37B—C36B—C41B	118.0 (2)
N1A—C3A—C4A	123.2 (2)	C26B—C36B—C37B	122.6 (2)
N1A—C5A—C10A	120.7 (2)	C26B—C36B—C41B	119.4 (2)
N1A—C5A—C6A	118.7 (2)	C36B—C37B—C38B	120.3 (2)
C6A—C5A—C10A	120.5 (2)	C37B—C38B—C39B	121.0 (3)
C5A—C6A—C7A	119.2 (3)	C38B—C39B—C40B	119.1 (3)
C6A—C7A—C8A	120.1 (3)	C39B—C40B—C41B	120.6 (3)
C7A—C8A—C9A	120.3 (3)	C36B—C41B—C40B	121.0 (3)
C8A—C9A—C10A	120.4 (3)	O2B—C42B—C27B	124.8 (2)
C5A—C10A—C9A	119.4 (3)	O2B—C42B—N8B	120.8 (2)
C1A—C11A—C12A	119.9 (2)	N8B—C42B—C27B	114.41 (19)
C1A—C11A—C16A	122.1 (2)	N8B—C43B—N9B	120.20 (19)
C12A—C11A—C16A	117.8 (2)	S2B—C43B—N9B	115.12 (16)
C11A—C12A—C13A	120.7 (2)	S2B—C43B—N8B	124.67 (17)
C12A—C13A—C14A	120.5 (3)	S2B—C44B—N10B	114.43 (17)
C13A—C14A—C15A	120.1 (3)	S2B—C44B—C45B	122.50 (18)
C14A—C15A—C16A	119.9 (3)	N10B—C44B—C45B	123.0 (2)
C11A—C16A—C15A	121.1 (2)	C44B—C45B—C46B	120.8 (2)
O1A—C17A—C2A	123.8 (2)	C46B—C45B—C50B	118.7 (2)
N3—C17A—C2A	116.2 (2)	C44B—C45B—C50B	120.5 (2)
O1A—C17A—N3	119.9 (2)	C45B—C46B—C47B	120.9 (3)
S1A—C18A—N4A	114.62 (17)	C46B—C47B—C48B	119.9 (3)
S1A—C18A—N3	124.69 (17)	C47B—C48B—C49B	119.9 (3)
N3—C18A—N4A	120.63 (19)	C48B—C49B—C50B	121.2 (3)
S1A—C19A—C20A	123.10 (17)	C45B—C50B—C49B	119.4 (3)
N5A—C19A—C20A	122.7 (2)	C28B—C29B—H38A	109.00
S1A—C19A—N5A	114.24 (18)	C28B—C29B—H38B	109.00
C21A—C20A—C25A	118.5 (2)	C28B—C29B—H38C	109.00
C19A—C20A—C25A	120.8 (2)	H38A—C29B—H38B	110.00
C19A—C20A—C21A	120.7 (2)	H38A—C29B—H38C	109.00
C20A—C21A—C22A	120.7 (2)	H38B—C29B—H38C	109.00
C21A—C22A—C23A	120.3 (3)	C30B—C31B—H40	120.00
C22A—C23A—C24A	119.9 (3)	C32B—C31B—H40	121.00
C23A—C24A—C25A	119.9 (2)	C31B—C32B—H41	120.00
C20A—C25A—C24A	120.7 (3)	C33B—C32B—H41	120.00
C3A—C4A—H6B	110.00	C32B—C33B—H42	120.00
C3A—C4A—H6A	110.00	C34B—C33B—H42	120.00
H6A—C4A—H6C	109.00	C33B—C34B—H43	120.00
C3A—C4A—H6C	110.00	C35B—C34B—H43	120.00
H6B—C4A—H6C	109.00	C30B—C35B—H44	120.00
H6A—C4A—H6B	109.00	C34B—C35B—H44	121.00
C5A—C6A—H8	120.00	C36B—C37B—H46	120.00
C7A—C6A—H8	120.00	C38B—C37B—H46	120.00
C6A—C7A—H9	120.00	C37B—C38B—H47	119.00
C8A—C7A—H9	120.00	C39B—C38B—H47	120.00
C9A—C8A—H10	120.00	C38B—C39B—H48	120.00
C7A—C8A—H10	120.00	C40B—C39B—H48	121.00
C10A—C9A—H11	120.00	C39B—C40B—H49	120.00
C8A—C9A—H11	120.00	C41B—C40B—H49	120.00
C9A—C10A—H12	120.00	C36B—C41B—H50	120.00
C5A—C10A—H12	120.00	C40B—C41B—H50	119.00
C11A—C12A—H14	120.00	C45B—C46B—H60	120.00
C13A—C12A—H14	120.00	C47B—C46B—H60	120.00
C14A—C13A—H15	120.00	C46B—C47B—H61	120.00
C12A—C13A—H15	120.00	C48B—C47B—H61	120.00
C13A—C14A—H16	120.00	C47B—C48B—H62	120.00
C15A—C14A—H16	120.00	C49B—C48B—H62	120.00
C14A—C15A—H17	120.00	C48B—C49B—H63	119.00
C16A—C15A—H17	120.00	C50B—C49B—H63	119.00
C11A—C16A—H18	119.00	C45B—C50B—H64	120.00
C15A—C16A—H18	120.00	C49B—C50B—H64	120.00
			
C19A—S1A—C18A—N3	176.2 (2)	C10A—C5A—C6A—C7A	−0.5 (4)
C19A—S1A—C18A—N4A	−0.97 (18)	C5A—C6A—C7A—C8A	1.3 (4)
C18A—S1A—C19A—N5A	0.81 (18)	C6A—C7A—C8A—C9A	−0.5 (5)
C18A—S1A—C19A—C20A	179.8 (2)	C7A—C8A—C9A—C10A	−1.2 (5)
C44B—S2B—C43B—N9B	0.46 (19)	C8A—C9A—C10A—C5A	1.9 (4)
C43B—S2B—C44B—N10B	−0.4 (2)	C1A—C11A—C12A—C13A	175.2 (3)
C44B—S2B—C43B—N8B	179.6 (2)	C12A—C11A—C16A—C15A	0.0 (4)
C43B—S2B—C44B—C45B	176.8 (2)	C16A—C11A—C12A—C13A	0.2 (4)
C3A—N1A—N2A—C1A	1.9 (3)	C1A—C11A—C16A—C15A	−174.9 (2)
C5A—N1A—N2A—C1A	−178.3 (2)	C11A—C12A—C13A—C14A	−0.2 (5)
N2A—N1A—C5A—C6A	−50.4 (3)	C12A—C13A—C14A—C15A	0.0 (5)
N2A—N1A—C5A—C10A	125.7 (2)	C13A—C14A—C15A—C16A	0.2 (5)
C3A—N1A—C5A—C6A	129.4 (3)	C14A—C15A—C16A—C11A	−0.2 (4)
C3A—N1A—C5A—C10A	−54.5 (3)	S1A—C19A—C20A—C25A	−14.8 (3)
N2A—N1A—C3A—C4A	177.5 (2)	N5A—C19A—C20A—C21A	−15.3 (3)
C5A—N1A—C3A—C2A	178.2 (2)	S1A—C19A—C20A—C21A	165.70 (19)
N2A—N1A—C3A—C2A	−2.1 (3)	N5A—C19A—C20A—C25A	164.1 (2)
C5A—N1A—C3A—C4A	−2.3 (4)	C21A—C20A—C25A—C24A	0.0 (3)
N1A—N2A—C1A—C2A	−1.0 (3)	C25A—C20A—C21A—C22A	−0.7 (4)
N1A—N2A—C1A—C11A	−176.1 (2)	C19A—C20A—C25A—C24A	−179.5 (2)
C18A—N3—C17A—O1A	4.2 (4)	C19A—C20A—C21A—C22A	178.8 (2)
C18A—N3—C17A—C2A	−172.8 (2)	C20A—C21A—C22A—C23A	1.0 (4)
C17A—N3—C18A—S1A	6.7 (3)	C21A—C22A—C23A—C24A	−0.5 (4)
C17A—N3—C18A—N4A	−176.3 (2)	C22A—C23A—C24A—C25A	−0.1 (4)
N5A—N4A—C18A—S1A	0.9 (2)	C23A—C24A—C25A—C20A	0.4 (4)
N5A—N4A—C18A—N3	−176.42 (19)	N7B—C26B—C27B—C28B	0.0 (3)
C18A—N4A—N5A—C19A	−0.3 (3)	N7B—C26B—C27B—C42B	179.6 (2)
N4A—N5A—C19A—S1A	−0.5 (2)	C36B—C26B—C27B—C28B	−176.1 (2)
N4A—N5A—C19A—C20A	−179.52 (19)	C36B—C26B—C27B—C42B	3.5 (4)
C28B—N6B—N7B—C26B	0.1 (3)	N7B—C26B—C36B—C37B	148.1 (2)
N7B—N6B—C28B—C29B	175.1 (2)	N7B—C26B—C36B—C41B	−31.5 (3)
C30B—N6B—N7B—C26B	177.6 (2)	C27B—C26B—C36B—C37B	−36.2 (4)
N7B—N6B—C28B—C27B	−0.1 (3)	C27B—C26B—C36B—C41B	144.3 (3)
N7B—N6B—C30B—C31B	−48.0 (3)	C26B—C27B—C28B—N6B	0.1 (3)
N7B—N6B—C30B—C35B	129.0 (3)	C26B—C27B—C28B—C29B	−174.7 (2)
C28B—N6B—C30B—C31B	129.1 (3)	C42B—C27B—C28B—N6B	−179.6 (2)
C28B—N6B—C30B—C35B	−54.0 (4)	C42B—C27B—C28B—C29B	5.7 (4)
C30B—N6B—C28B—C27B	−177.3 (2)	C26B—C27B—C42B—O2B	59.4 (4)
C30B—N6B—C28B—C29B	−2.1 (4)	C26B—C27B—C42B—N8B	−122.2 (3)
N6B—N7B—C26B—C27B	0.0 (3)	C28B—C27B—C42B—O2B	−121.1 (3)
N6B—N7B—C26B—C36B	176.5 (2)	C28B—C27B—C42B—N8B	57.4 (3)
C42B—N8B—C43B—S2B	14.2 (3)	N6B—C30B—C31B—C32B	176.1 (3)
C43B—N8B—C42B—C27B	178.2 (2)	C35B—C30B—C31B—C32B	−0.8 (4)
C42B—N8B—C43B—N9B	−166.7 (2)	N6B—C30B—C35B—C34B	−175.8 (3)
C43B—N8B—C42B—O2B	−3.3 (4)	C31B—C30B—C35B—C34B	1.1 (4)
N10B—N9B—C43B—S2B	−0.5 (3)	C30B—C31B—C32B—C33B	−0.6 (5)
N10B—N9B—C43B—N8B	−179.7 (2)	C31B—C32B—C33B—C34B	1.7 (5)
C43B—N9B—N10B—C44B	0.2 (3)	C32B—C33B—C34B—C35B	−1.4 (6)
N9B—N10B—C44B—S2B	0.2 (3)	C33B—C34B—C35B—C30B	0.1 (5)
N9B—N10B—C44B—C45B	−176.9 (2)	C26B—C36B—C37B—C38B	−178.2 (2)
N2A—C1A—C2A—C3A	−0.2 (3)	C41B—C36B—C37B—C38B	1.3 (4)
N2A—C1A—C2A—C17A	174.5 (2)	C26B—C36B—C41B—C40B	178.3 (3)
C11A—C1A—C2A—C3A	174.1 (2)	C37B—C36B—C41B—C40B	−1.3 (5)
C2A—C1A—C11A—C12A	154.8 (3)	C36B—C37B—C38B—C39B	−0.8 (4)
C2A—C1A—C11A—C16A	−30.5 (4)	C37B—C38B—C39B—C40B	0.1 (5)
N2A—C1A—C11A—C16A	143.4 (2)	C38B—C39B—C40B—C41B	0.0 (5)
C11A—C1A—C2A—C17A	−11.2 (4)	C39B—C40B—C41B—C36B	0.6 (6)
N2A—C1A—C11A—C12A	−31.3 (3)	S2B—C44B—C45B—C46B	−2.4 (4)
C1A—C2A—C3A—C4A	−178.2 (2)	S2B—C44B—C45B—C50B	178.7 (2)
C1A—C2A—C17A—O1A	147.2 (3)	N10B—C44B—C45B—C46B	174.4 (3)
C17A—C2A—C3A—N1A	−174.0 (2)	N10B—C44B—C45B—C50B	−4.5 (4)
C17A—C2A—C3A—C4A	6.6 (4)	C44B—C45B—C46B—C47B	−178.2 (3)
C1A—C2A—C3A—N1A	1.3 (2)	C50B—C45B—C46B—C47B	0.7 (5)
C3A—C2A—C17A—O1A	−38.9 (4)	C44B—C45B—C50B—C49B	178.3 (3)
C3A—C2A—C17A—N3	138.0 (2)	C46B—C45B—C50B—C49B	−0.6 (5)
C1A—C2A—C17A—N3	−35.9 (4)	C45B—C46B—C47B—C48B	−0.8 (5)
N1A—C5A—C6A—C7A	175.6 (2)	C46B—C47B—C48B—C49B	0.9 (6)
N1A—C5A—C10A—C9A	−177.1 (2)	C47B—C48B—C49B—C50B	−0.8 (5)
C6A—C5A—C10A—C9A	−1.0 (4)	C48B—C49B—C50B—C45B	0.7 (5)

Symmetry codes: (i) −*x*+2, −*y*+1, −*z*; (ii) −*x*+2, −*y*, −*z*+1; (iii) *x*, *y*+1, *z*; (iv) *x*−1, *y*, *z*; (v) −*x*+1, −*y*+1, −*z*+1; (vi) *x*−1, *y*+1, *z*; (vii) −*x*+3, −*y*+1, −*z*; (viii) *x*+1, *y*, *z*; (ix) −*x*+3, −*y*, −*z*; (x) *x*+1, *y*−1, *z*; (xi) −*x*+2, −*y*, −*z*; (xii) *x*, *y*−1, *z*.

### Hydrogen-bond geometry (Å, º)

**Table:**

*D*—H···*A*	*D*—H	H···*A*	*D*···*A*	*D*—H···*A*
N3—H21···N9*B*	0.86	2.13	2.916 (3)	151
N8*B*—H53···N4*A*	0.86	2.07	2.902 (3)	163
C4*A*—H6*B*···O1*A*	0.96	2.42	3.107 (3)	128
C29*B*—H38*C*···N5*A*	0.96	2.59	3.443 (3)	148
C37*B*—H46···O2*B*	0.93	2.41	3.184 (3)	141
